# Clinical and neonatal outcomes of patients of different ages following transfer of thawed cleavage embryos and blastocysts cultured from thawed cleavage-stage embryos

**DOI:** 10.1371/journal.pone.0207340

**Published:** 2018-11-26

**Authors:** Qin-Wei Zhou, Shuang Jing, Li Xu, Hui Guo, Chang-Fu Lu, Fei Gong, Guang-Xiu Lu, Ge Lin, Yi-Fan Gu

**Affiliations:** 1 Institute of Reproductive and Stem Cell Engineering, School of Basic Medical Science, Central South University, Changsha City, Hunan Province, China; 2 Reproductive & Genetic Hospital of CITIC-XIANGYA, Changsha City, Hunan Province, China; 3 National Engineering and Research Center of Human Stem Cell, Changsha City, Hunan Province, China; 4 Key Laboratory of Reproductive and Stem Cell Engineering, National Health and Family Planning Commission, Changsha City, Hunan Province, China; Utah State University, UNITED STATES

## Abstract

**Background:**

Frozen-thawed embryo transfer (FET) has become a routine procedure in assisted reproductive technology (ART). In FET, although blastocysts cultured from thawed cleavage-stage embryos are associated with better perinatal outcomes. it may increase cycle cancellation due to no suitable embryo to transfer. The overall clinical outcomes following transfer of thawed cleavage-stage FET and blastocysts cultured from thawed cleavage-stage embryos in young and advanced age patients remains unclear. Therefore, we aimed to identify the optimal FET strategy in young and advanced age women who undergo FET.

**Methods:**

This retrospective study included 16,387 thaw cycles. We retrospectively analyzed data of couples who had completed the first FET cycle. Two FET strategies were studied: transfer of thawed cleavage-stage embryos (strategy A) or blastocysts cultured from thawed cleavage-stage embryos (strategy B). The clinical and neonatal outcomes of two FET strategies were compared in young (<35 years) and advanced (≥35 years) age women.

**Results:**

In young women, the clinical outcomes per transfer cycle were better in strategy B than strategy A. While the clinical pregnancy (59.29%, 52.60%) and live birth rates (49.37%, 43.88%) per thaw cycle were significantly higher in strategy A than in B. In women of advanced age, the clinical outcomes per transfer cycle were still better in strategy B than in A, and the clinical pregnancy (36.44%, 39.66%) and live birth rates (25.70%, 30.00%) per thaw cycle were significantly higher in strategy B than in A.

**Conclusions:**

FET of blastocysts cultured from cleavage-stage embryos showed higher efficiency for per transfer cycle whether in younger or advanced age women. Whereas, when cycle cancellations due to no suitable embryo to transfer were considered, cleavage-stage FET was found to be more suitable for younger women, while FET of blastocysts cultured from cleavage-stage embryos was better suited for women of advanced age.

## Introduction

Since the first successful case of clinical pregnancy established using frozen embryos in the 1980s, frozen-thawed embryo transfer (FET) has become a routine procedure in assisted reproductive technology (ART) [1]. Cryopreservation techniques and the developmental potential of frozen-thawed embryos have considerably improved since then, and the clinical pregnancy rates and some clinical outcomes associated with FET are even higher than those of fresh embryos [2]. There were no safety issues in children born from ART using frozen cycles compared to those born from ART using fresh cycles [3].

To date, FET at the cleavage stage is still widely used, especially in those large ART centers, because this strategy may reduce the requirement for incubators, schedule culture time more flexibly and facilitate the management of more patient cycles under reasonable workloads [4]. Whereas, the implantation and clinical pregnancy rates after blastocyst transfer have been demonstrated to be superior to cleavage-stage embryo transfer [5]. In addition, blastocyst-stage transfer can improve both embryonic and uterine synchronicity and self-selection of viable embryos, which resulted in higher live birth rates in fresh cycles [6]. Wang et al. [7, 8] suggested that blastocysts that have been cultured from thawed cleavage-stage embryos are associated with better perinatal outcomes. Eftekhar et al. [9] reported that transferring blastocyst-stage embryos derived from thawed cleavage-stage embryos improved the ongoing pregnancy rate.

These studies above have some limitations: First, these studies did not follow the live birth and neonatal outcomes. Second, the clinical outcomes per thaw cycle were not compared, because blastocyst culture increases the overall cycle cancellation due to unavailability of blastocysts for transfer [10, 11], which may introduce bias in clinical outcomes per transfer cycle. Finally, only few of these studies have investigated the optimal embryo transfer strategy for women of different age groups. Therefore, the ideal strategy following a transfer from thawed cleavage-stage and blastocyst-stage embryos cultured from thawed cleavage-stage embryos resulting in better overall clinical and neonatal outcomes for women of young and advanced age remains to be determined [12–14].

As we know, maternal age is the most significant factor affecting clinical outcomes in ART [15, 16]. In a previous study on women between 35–39 years of age and ≥40 years of age reported that women in these two age groups had 51% and 177% increased risks of miscarriage, respectively, relative to women below years [8]. Wang et al. [17] also showed that women of advanced age had lower implantation and pregnancy rates than young women. These studies implied that different clinical strategies should be employed depending on the age of the woman.

In this study, we approached the above-mentioned problems by studying the clinical and perinatal outcomes of young patients (<35 years) and advanced age patients (≥35 years). Our study aims to identify a suitable FET strategy for infertile women who undergo frozen cleavage-stage embryo transfer associated with the best outcomes.

## Materials and methods

### Patient selection and study design

In this retrospective cohort study, we traced infertile couples who had completed the first FET cycle after fresh cycles from January 2010 to December 2015. The inclusion criteria were as follows: 1) maternal age, 20–42 years; 2) ≥3 frozen embryos; 3) frozen embryos are at the cleavage stage; 4) the first FET cycle after a previous fresh cycle. The patients were screened according to the following exclusion criteria: 1) oocyte donation cycles; 2) mixed cleavage-/blastocyst-stage embryo transfer cycles; 3) preimplantation genetic diagnostic/preimplantation genetic screening (PGD/PGS) cycles. In this study, we included papers involving two FET strategies: thawed cleavage-stage ET (strategy A) or blastocysts cultured from thawed cleavage-stage embryos (strategy B). The clinical and neonatal outcomes of these two FET strategies were compared in young (<35 years) and advanced age (≥35 years) women. In our study, the thawed embryo transfer strategy to be used was decided depending on the patients’ decision. Before FET, we spoke with the patients who froze their embryos at the cleavage stage and informed them of the possible circumstances when transferring cleavage-stage embryos or blastocysts cultured from thawed cleavage-stage embryos.

**Fig 1:**
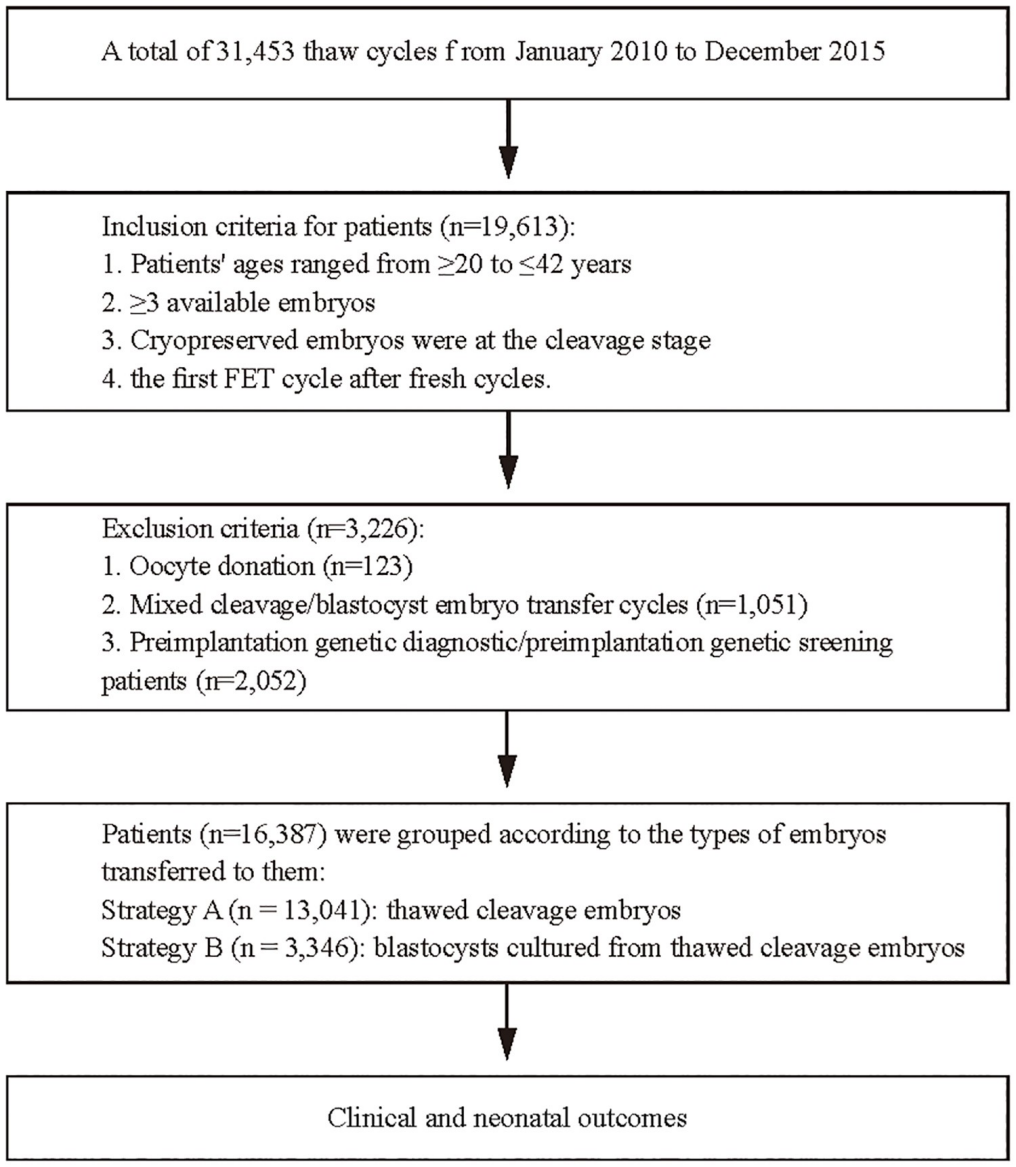
Flowchart of study design.

This retrospective study included 16,387 embryo frozen-thawed cycles, in which 15,408 cycles accomplished embryo transfer, and 979 cycles were cancelled due to no suitable embryos to transfer. Fig 1 presents a flowchart illustrating the study design. This study protocol and study design were approved by the Ethics Committee of the CITIC-Xiangya Reproductive & Genetic Hospital (LL-SC-SG-2012-015). All patient data were fully anonymized before access, and the ethics committee waived the requirement for written informed consent to have data from their medical records used in research.

### Controlled ovarian stimulation

Ovarian stimulation was carried out using a long luteal gonadotropin-releasing hormone (GnRH) agonist protocol or an antagonist protocol, as described previously[18], Briefly, once two-thirds of the follicles reached 18 mm, 5,000–10,000 IU of human chorionic gonadotropin (hCG; Pregnyl, Merck) was injected. At 35–36 h after hCG administration, oocytes were collected in G-IVF media (Vitrolife) under transvaginal ultrasound guidance.

### Embryo culture and grading

All oocytes were fertilized by in vitro fertilization (IVF) or intracytoplasmic sperm injection (ICSI) 4–6 h after oocyte retrieval. Normal fertilization was assessed 16–18 h after insemination or injection. The resultant embryos were cultured in G1.5 medium (Vitrolife) until they reached cleavage stage. All oocytes, zygotes, and embryos were cultured at 37°C in an atmosphere containing 6% CO_2_, 5% O_2_, and 89% N_2_.

Cleavage-stage embryos were evaluated on day 3 after oocyte retrieval according to the appearance of blastomeres and the percentage of fragments on the basis of conventional criteria [19]. Embryos that had reached the 4-cell stage or higher and with a percentage of embryo fragments not exceeding 20% were considered suitable for transfer or cryopreservation. Cleavage embryos were defined as high-quality in this study if the following criteria were met: i) normal fertilization; ii) at least six blastomeres; iii) the blastomere size is stage-specific; iv) the percentage of embryo fragments does not exceed 10%; v) the blastomere is transparent without cytoplasmic inclusions or vacuoles, vi) there are no multinucleated blastomeres. Embryos were generally cryopreserved at the early cleavage stage on day 3 in this study.

### Freezing and thawing protocol

Cleavage-stage embryos were vitrified using a Kitazato vitrification kit (Kitazato Biopharma) using high-security vitrification straws (Cryo Bio System). Embryos were vitrified and thawed according to the manufacturer’s instructions. Cleavage-stage embryos were thawed using commercially available warming solution (Kitazato Biopharma), following the manufacturer’s instructions.

### Selection of embryos for FET

In strategy A, two high-quality cleavage-stage embryos were selected for thawing and culture in G2.5 media (Vitrolife) for 1–2 h. Embryos were considered to be survived and suitable for transfer if at least half of their blastomeres were intact after culture. In strategy B, all cleavage-stage embryos were thawed and the survived embryos were cultured in G2.5 media (Vitrolife) up to day 5. Blastocyst morphology was evaluated on day 5 according to the criteria described by Gardner and Schoolcraft. Briefly, blastocysts were numerically scored on a scale of 1 to 6 based on their expansion stage and hatching status as follows: 1, an early blastocyst with a blastocoel that is less than half of the volume of the embryo; 2, a blastocyst with a blastocoel that is half of or greater than half of the volume of the embryo; 3, a full blastocyst with a blastocoel completely filling the embryo; 4, an expanded blastocyst with a blastocoel volume larger than that of the early embryo, with a thinning zona; 5, a hatching blastocyst with the trophectoderm starting to herniate though the zona; and 6, a hatched blastocyst, in which the blastocyst has completely escaped from the zona. Inner cell mass (ICM) development was scored as follows: A, tightly packed, many cells; B, loosely grouped, several cells; or C, very few cells. The trophectoderm (TE) was scored as follows: A, many cells forming a cohesive epithelium; B, few cells forming a loose epithelium; or C, very few large cells. For example, a 4AB blastocyst means blastocyst stage = 4, ICM grade = A and TE grade = B. Blastocysts that reached ≥3 BC (blastocyst stage = 3, 4, 5, or 6; ICM grade = A or B; TE grade = A, B or C) were considered suitable for transfer, and those that reached ≥4 BB (blastocyst stage = 4, 5, or 6; ICM grade = A or B; TE grade = A or B) were considered to be of high quality. Only one high-quality blastocyst or at most two suitable blastocysts were transferred per FET cycle. The remaining suitable blastocysts were frozen again.

### FET and luteal support

Cleavage-stage embryos and blastocysts were transferred 3–5 days after ovulation in a natural cycle or 3–5 days after the start of progesterone therapy with an endometrial preparation containing estradiol valerate and progesterone. Briefly, 6 mg of estradiol valerate was administered from day 3 for 10–15 days, and luteal support was given when the endometrial thickness was confirmed to be ≥8 mm by ultrasound examination.

### Outcome measures and follow-up

The implantation rate was calculated as the ratio of the number of observed embryo heartbeats to the number of transferred embryos. Clinical pregnancy was confirmed by an ultrasound conducted 4 weeks after the ET. The clinical pregnancy rate per transfer cycle was calculated as the number of clinical pregnancy cycles to the number of ET cycles. The pregnancy rate per thaw cycle was the number of clinical pregnancy cycles divided by the number of thaw cycles. The live birth rate per ET cycle was defined as the number of deliveries resulting in live births divided by the number of ET cycles. The live birth rate per thaw cycle was the number of deliveries resulting in live births cycles divided by the number of thaw cycles, delivery of a multiple pregnancy was considered a single live birth. The early/late miscarriage rate was calculated as the number of early/late miscarriage cycles to the number of clinical pregnancy cycles. Gestational age was defined as the number of completed weeks of gestation and calculated using the formula “(pregnancy end date—embryo transfer date) + 16 days” for cleavage embryo transfer and “(pregnancy end date—embryo transfer date) + 19 days” for blastocyst transfer.

Preterm and post-term births were defined as deliveries before 37 or after 42 completed weeks of gestation. Neonates were categorized as normal (2,500–4,000 g), low birth weight (<2,500 g), and macrosomia (>4,000 g) according to their birth weight. Couples were contacted by phone to obtain neonatal information, including their dates of birth, birth weight, gender, birth defects, and neonatal diseases, as a part of the routine follow-up checks.

### Statistical analysis

Data were statistically analyzed using Statistical Package for Social Sciences software version 19.0 (SPSS). Before analyses, continuous variables were analyzed using a normality test. Normally distributed data were expressed as means ± SD, and data with non-normal distribution were expressed as median (1^st^ to 3^rd^ quartile). Differences between the strategies were compared using the independent Student’s *t* test in normally distributed data including the body mass index (BMI). Non-normally distributed data (age, EM thickness, gestational age, and birth weight) were compared using the Mann-Whitney test. Categorical variables were presented as percentages and analyzed using the χ^2^ test. Fisher’s exact test was used if necessary. Categorical variables included the BMI category, number of transferred embryos, gestational age, birth weight, infertility types, endometrial preparation, rates of clinical outcomes, number of newborns, gender of newborns, perinatal deaths, and birth defects. Statistical significance was set at <0.05.

Logistic regression analysis was used to control for potential confounders, including age, infertility types, endometrial preparation, and endometrial thickness, and to evaluate any potential effects (strategy A vs. strategy B in young women and advanced age women).

## Results

### Patient characteristics

A total of 16,387 frozen-thawed embryo cycles were incorporated in the study, including 15,408 embryo transfer cycles and 979 cancelled cycles in these transfer cycles. A total of 30,002 embryos were transferred. Patients who perform cryopreservation embryo transfer were divided into thawed cleavage-stage embryo transfer (Strategy A, n = 13,041), and transfer of blastocysts cultured from thawed cleavage-stage embryos (Strategy B, n = 3,346).

Statistical analysis revealed that there were no differences in the BMI between the two strategies in women of young and advanced age (Table 1). However, there were significant differences in the age, infertility types, endometrial preparation (EM), endometrial thickness, and number of embryos transferred using the two strategies in young and advanced age women (p < 0.05).

**Table 1:** Patient characteristics.

	Young women (<35 years)			Advanced age women (≥35 years)		
	Strategy A	Strategy B	P value	Strategy A	Strategy B	P value
**No. of thaw cycles**	10,146	2,466		2,895	880	
**No. of ET cycles**	9,985	1,996		2,768	659	
**Age (years) (range)**	29 (27–32)	30 (27–32)	<0.001	37 (36–39)	37 (36–39)	0.124
**BMI (kg/m**^**2**^**) (mean ± SD)**	21.37±2.72	21.40±2.71	0.608	22.36±2.60	22.43±2.59	0.485
**<18.5 (%)**	1,319 (13.00)	313 (12.69)	0.918	142 (4.91)	38 (4.32)	0.387
**18.5–24.99 (%)**	7,834 (77.21)	1,912 (77.53)		2,313 (79.90)	693 (78.75)	
**≥25 (%)**	993 (9.79)	241 (9.77)		440 (15.20)	149 (16.93)	
**Infertility types**						
**Primary (%)**	5,189 (51.14)	1,193 (48.38)	0.014	627 (21.66)	233 (26.48)	0.003
**Secondary (%)**	4,957 (48.86)	1,273 (51.62)		2,268 (78.34)	647 (73.52)	
**EM preparation**						
**NC (%)**	3,151 (31.06)	447 (18.13)	<0.001	1,001 (34.58)	178 (20.23)	<0.001
**HRT (%)**	6,995 (68.94)	2,019 (81.87)		1,894 (65.42)	702 (79.77)	
**EM thickness (mm) (range)**	10.9 (10.0–12.0)	10.7 (9.7–11.9)	<0.001	10.7 (9.8–11.9)	10.5 (9.6–11.8)	0.001
**No. of transferred embryos (range)**	2 (2–2)	2 (1–2)		2 (2–2)	2 (0–2)	
**SET** (%)	62 (0.62)	524 (26.25)	<0.001	46 (1.66)	182 (27.62)	<0.001
**DET** (%)	9,923 (99.38)	1,472 (73.75)		2,722 (98.34)	477 (72.38)	

Note: Strategy A: transfer of thawed cleavage-stage embryos; Strategy B: transfer of blastocysts cultured from thawed cleavage-stage embryos; ET: embryo transfer; BMI: body mass index; EM: endometrium; NC: natural cycle; HRT: hormone replacement treatment; SET: single embryo transfer cycles; DET: double embryo transfer cycles

### Clinical outcomes

Table 2 illustrates the clinical outcomes of both patient groups. In young women, a total of 23,376 embryos were transferred in 11,981 cycles, and 9,922 embryos were implanted (42.45%). In advanced age women, 6,626 embryos were transferred in 3,427 cycles, whereas only 1,735 embryos were implanted (26.18%).

**Table 2:** Comparison of clinical outcomes between strategies A and B in women of young and advance age undergoing FET.

	Young women (<35 years)			Advanced age women (≥35 years)		
	Strategy A	Strategy B	P value	Strategy A	Strategy B	P value
**No. of thaw cycles**	10,146	2,466		2,895	880	
**No. of ET cycles**	9,985	1,996		2,768	659	
**No. of canceled cycles**	161 (1.59)	470 (19.06)	<0.001	127 (4.39)	221 (25.11)	<0.001
**No. of transferred embryos**	19,908	3,468		5,490	1,136	
**Implantation**	8,195 (41.16)	1,727 (49.80)	<0.001	1,295 (23.59)	440 (38.73)	<0.001
**Clinical pregnancy**	6,016	1,297		1,055	349	
**per ET cycle** (%)	(60.25)	(64.98)	<0.001	(38.11)	(52.96)	<0.001
**per thaw cycle** (%)	(59.29)	(52.60)	<0.001	(36.44)	(39.66)	0.084
**Multiple conception** (%)	1,511 (25.12)	297 (22.90)	0.093	135 (12.80)	49 (14.04)	0.551
**Miscarriage** (%)	822 (13.66)	200 (15.42)	0.097	284 (26.92)	80 (22.92)	0.140
**≤12 weeks** (%)	606 (10.07)	147 (11.33)	0.175	234 (22.18)	64 (18.34)	0.128
**> 12 weeks** (%)	216 (3.59)	53 (4.09)	0.389	50 (4.74)	16 (4.58)	0.906
**Ectopic pregnancy**	197 (3.27)	17 (1.31)	<0.001	27 (2.56)	5 (1.43)	0.222
**Live birth**	5,009	1,082		744	264	
**per ET cycle** (%)	(50.17)	(54.21)	0.001	(26.88)	(40.06)	0.001
**per thaw cycle** (%)	(49.37)	(43.88)	<0.001	(25.70)	(30.00)	0.012

Note: Strategy A: transfer of thawed cleavage-stage embryos; Strategy B: transfer of blastocysts cultured from thawed cleavage-stage embryos; ET: embryo transfer.

In young women, the implantation rate (41.16% vs. 49.80%, P < 0.001, all were strategy A vs. B, respectively), clinical pregnancy rate (60.25% vs. 64.98%, P < 0.001) and live birth rate (50.17% vs. 54.21%, P = 0.001) in per transfer cycle were significantly higher in strategy B than strategy A. The number of cycles that had been canceled was significantly higher in strategy B (1.59% vs. 19.06%, P < 0.001) than in strategy A. The ectopic pregnancy rate was significantly lower in strategy B than in strategy A (3.27% vs. 1.31%, P < 0.001). In all the thaw cycles, the clinical pregnancy (59.29% vs. 52.60%, P < 0.001) and live birth rates were significantly lower in strategy B (49.37% vs. 43.88%, P < 0.001) than in strategy A.

In advanced age women, the implantation (23.59% vs. 38.73%, P < 0.001), clinical pregnancy (38.11% vs. 52.96%, P < 0.001), and live birth rates per transfer cycle (26.88% vs. 40.06%, P < 0.001) were significantly higher in strategy B than in strategy A. There was a higher percentage of canceled cycles in strategy B (4.39% vs. 25.11%, P < 0.001) than in strategy A. Although the clinical pregnancy per thaw cycle (36.44% vs. 39.66%, P = 0.084) was higher in strategy B than strategy A, it was not statistically significant. However, the live birth rate (25.70% vs. 30.00%, P = 0.012) was still significantly higher in strategy B than in strategy A for all thaw cycles.

### Logistic regression

Table 3 presents the logistic regression analysis for the live birth rate per thaw cycle. In young women, upon controlling for potential confounders, the live birth rate (AOR 1.19, 95% CI 1.09–1.31, *p* < 0.001) was significantly higher in strategy A than in strategy B. In advanced age women, the live birth rate (AOR 0.79 95% CI 0.67–0.94, *p* = 0.008) was significantly higher in strategy B than in strategy A.

**Table 3:** Regression analysis for the live birth rate per thaw cycle.

	Young women (<35 years)			Advanced age women (≥35 years)		
	OR(95%CI)	AOR(95%CI)	P value	OR(95%CI)	AOR(95%CI)	P value
**Group**						
**Strategy A**	1.25 (1.14–1.36)	1.19 (1.09–1.31)	<0.001	0.81 (0.68–0.95)	0.79 (0.67–0.94)	0.008
**Strategy B**	Reference			Reference		
**Female age**	0.96 (0.94–0.97)	0.96 (0.95–0.97)	<0.001	0.83 (0.80–0.86)	0.83 (0.80–0.86)	<0.001
**BMI**	0.99 (0.97–1.00)	0.99 (0.98–1.01)	0.346	1.01 (0.98–1.03)	1.02 (0.99–1.05)	0.146
**Infertility types**						
**Primary**	1.21 (1.13–1.29)	1.12 (1.04–1.21)	0.002	1.05 (0.89–1.25)	0.95 (0.79–1.13)	0.525
**Secondary**	Reference			Reference		
**EM preparation**						
**NC**	1.21 (1.12–1.31)	1.15 (1.06–1.25)	0.001	1.24 (1.07–1.45)	1.21 (1.03–1.42)	0.022
**HRT**	Reference			Reference		
**EM thickness (mm)**	1.07 (1.05–1.10)	1.06 (1.03–1.08)	<0.001	1.06 (1.01–1.10)	1.05 (1.01–1.09)	0.021

Note: Strategy A: transfer of thawed cleavage-stage embryos; Strategy B: transfer of blastocysts cultured from thawed cleavage-stage embryos; OR: odds ratio; AOR: adjusted odds ratio; BMI: body mass index; EM: endometrium; NC: natural cycle; HRT: hormone replacement treatment

### Neonatal outcomes

Table 4 presents the neonatal outcomes between strategy A and strategy B among young women and advanced age women. There were no significant differences in the percentages of singleton and twin births, perinatal deaths, and birth defects between strategies A and B among young women undergoing FET. The median gestational age was significantly higher in strategy A than B (38.5 vs. 38.4 weeks, P < 0.001), the median birth weights was significantly higher in strategy B than in A (3.0 vs. 3.1 kg, P = 0.011).

**Table 4:** Characteristics of newborns born to women of young and advance age who underwent FET via strategies A and B.

	Young women (<35 years)			Advanced age women (≥35 years)		
	Strategy A	Strategy B	P value	Strategy A	Strategy B	P value
**No. of newborns**	6,520	1,379		879	313	
**Singleton (%)**	3,498 (69.83)	785 (72.55)	0.076	609 (81.85)	215 (81.44)	0.881
**Twin (%)**	1,511 (30.17)	297 (27.45)		135 (18.15)	49 (18.56)	
**Gender of newborns**						
**Male (%)**	3,343 (51.27)	789 (57.22)	<0.001	462 (52.56)	176 (56.23)	0.264
**Female (%)**	3,177 (48.73)	590 (42.78)		417 (47.44)	137 (43.77)	
**Gestational age (weeks)(range)**	38.5 (37.4–39.5)	38.4 (37.1–39.4)	<0.001	38.6 (37.4–39.4)	38.4 (37.4–39.2)	0.058
**37~42 wk (%)**	4,207 (83.99)	870 (80.41)	0.013	632 (84.95)	222 (84.09)	0.256
**<37 wk (%)**	788 (15.73)	210 (19.41)		112 (15.05)	41 (15.53)	
**>42 wk (%)**	14 (0.28)	2 (0.18)		0 (0.00)	1 (0.38)	
**Birth weight (kg) (range)**	3.0 (2.6–3.4)	3.1 (2.6–3.5)	0.011	3.2 (2.7–3.6)	3.2 (2.8–3.6)	0.251
**2.5~4.0 kg (%)**	5106 (78.31)	1060 (76.87)	0.018	710 (80.77)	256 (81.79)	0.836
**<2.5 kg (%)**	1199 (18.39)	252 (18.27)		121 (13.77)	39 (12.46)	
**>4.0 kg (%)**	215 (3.30)	67 (4.86)		48 (5.46)	18 (5.75)	
**Perinatal deaths (%)**	31 (0.48)	9 (0.65)	0.400	7 (0.80)	3 (0.96)	0.728
**Birth defects (%)**	133 (2.04)	30 (2.18)	0.748	14 (1.59)	6 (1.92)	0.701

Note: Strategy A: transfer of thawed cleavage-stage embryos; Strategy B: transfer of blastocysts cultured from thawed cleavage-stage embryos.

There were no significant differences in the percentages of singleton and twin births, gender, gestational age, birth weights, perinatal deaths, and neonatal birth defects between strategies A and B among women of advanced age undergoing FET.

## Discussion

In this retrospective cohort study, we compared two frozen embryo transfer strategies between young and advanced age women: transfer of thawed cleavage embryos or blastocyst cultured from thawed cleavage-stage embryos. The results of our study revealed that FET of blastocysts cultured from cleavage-stage embryos showed higher efficiency for each transfer cycle regardless of patient age group. When cycle cancellations due to no suitable embryo to transfer were accounted for, cleavage-stage FET was found to be more suitable for younger women than advanced age women, while FET of blastocysts cultured from cleavage-stage embryos was better suited for women of advanced age.

Logistic regression analysis was used to determine whether the live birth rate per thaw cycle is related to independent variables, such as the different transfer strategies used, female age, BMI, infertility types (primary or secondary), EM preparation, and EM thickness in young women and advanced age women. Upon controlling for potential confounders, these differences still exist among the two strategies in young and advanced age women.

Some studies reported improved prenatal outcomes following fresh blastocyst transfer [20, 21] and transfer of blastocysts from thawed cleavage-stage embryos [7]. A prospective study also showed that blastocyst formation from thawed cleavage-stage embryos can indicate embryo viability and pregnancy outcome well [9]. Blastocyst transfer has been found to be associated with higher live birth rates in fresh cycles by improving embryo-uterine synchronicity and self-selection of viable embryos [6]. However, embryos transferred at the blastocyst stage tend to have a higher proportion of patients with no viable embryos, resulting in a higher overall cycle cancellation rate than those transferred at the cleavage stage [10, 11, 22]. Some studies have suggested that the blastocyst formation rate decreases as maternal age increases [23–25]. Blastocyst culture may result in high cycle cancellation rate in advanced age women with poor ovarian reserve [26, 27]. It was obvious that the blastocyst transfer had a significantly higher cancellation rate than cleavage-stage embryo transfer due to no suitable embryo to transfer. This result implies that although the efficiency of blastocyst-stage transfer in per transfer cycle is high, but it may not be the case in per thaw cycles.

Although blastocyst culture of frozen-thawed embryos was found to be associated with better outcomes per transfer cycle, patients may not benefit from it if the cancellation rate is high. Thus, in order to reach a balance between cancellation and clinical outcomes, the clinical outcomes per thaw cycle were supplemented and the live births rate per thaw cycles were considered as the ultimate standard; these parameters were also utilized in other studies [28, 29]. Similar clinical outcomes per oocyte retrieval or per initiated cycle, including the cancelled cycles, have been calculated in these studies [30, 31]. Kato et al. [32] considered that estimating the “per oocyte retrieval” success rate would lead to minimal bias. However, their clinical outcomes in total cycles were similar to the per transfer cycle between different groups. Our study exhibited different results, which may be a consequence of the difference between young and advanced age women.

There is a well-established link between reduced reproductive potential and advanced maternal age [33]. This natural decrease in advanced age women results from several factors, including decreased oocyte quantity and embryo quality [34]. Also, the mitochondrial DNA copy number in oocytes was significantly lower in older women than in younger women, influencing embryonic development and pregnancy outcome [35]. More importantly, Battaglia et al. [36] reported that meiotic spindle assembly was significantly altered in women of advanced age, resulting in a high prevalence of aneuploidy in oocytes. Minasi et al. [37]reported the mean maternal age was lower in euploid strategies compared to the aneuploid strategies. At least 10% of human embryos were found to be aneuploid, and aneuploidy was present in up to 50% of embryos in advanced age women [38]. On the other hand, transferring embryos at the blastocyst stage will decrease the probability of aneuploid embryo transfer, consequently increasing the likelihood of pregnancy [14]. Natural selection would occur following culture for several days, allowing a reduction in the number of aneuploid embryos. Consequently, blastocyst culture will enhance the possibility of self-selection of cleavage embryos [39], especially in the case of advanced age women who are at a greater risk of aneuploid embryos. The advantage of self-selection in blastocysts may be incompletely demonstrated in young women due to fewer aneuploid embryos. Therefore, blastocyst culture is more suitable for women of advanced age.

A recent study suggested that blastocyst transfer or blastocyst culture was still suitable to advanced age patients [40]. Cycle cancellation does not mean no gain. Embryos that fail to reach the blastocyst stage are likely to be of poor quality. The outcome may not improve even if the embryos are transferred at the cleavage stage. Chen et al. [40] concluded that blastocyst culture will not adversely affect the pregnancy outcomes of advanced age women with low ovarian reserve. Preimplantation genetic testing for aneuploidy has been recommended for such women with a limited number of embryos [41].

Apart from achieving successful implantation and pregnancy, ART focuses on producing a healthy baby. Therefore, we compared neonatal outcomes between the two strategies in young and advanced age women. Consistent with the results of previous studies [42–45], young women who received blastocyst transfer gave birth to more boys than young women who received cleavage-stage embryo transfer; one possible reason is that male embryos exhibit faster cleavage, consequently influencing blastocyst selection for transfer [46]. In young women, the average birth weight of newborns after blastocyst-stage embryo transfer was significantly higher than that after cleavage-stage transfer, these could be induced by the influence of epigenetic alteration on the birthweight of newborns during blastocyst culture [47]. Consistent with this finding, blastocyst transfer has been found to be associated with a higher risk of preterm delivery than cleavage-stage embryo transfer, some studies have also shown that male gender is associated with preterm deliveries, the precise reasons are unclear [48–50].

The strengths of this study include its large sample size, clinical outcome per thaw cycle, and neonatal outcomes. However, these results should be approached with caution due to the limitations of this study, primarily its retrospective design and confounding factors. In future, randomized controlled trials with a large sample size should be carried out to verify the findings of this study. This study validates existing data and offers useful information that can be used to counsel patients undergoing FET to select a suitable strategy to improve their chances of a healthy and safe pregnancy and birth of a healthy child.

## Conclusions

In conclusion, FET of blastocysts cultured from cleavage-stage embryos showed higher efficiency per transfer cycle whether in younger or advanced age women. However, if cycle cancellations due to no suitable embryo for transfer were accounted for, cleavage-stage FET was found to be more suitable for younger women, while FET of blastocysts cultured from cleavage-stage embryos was better suited for women of advanced age.

## Supporting information

S1 File PLOSOne_Clinical_Studies_Checklist. (DOCX)

S2 File STROBE_checklist_v4_combined_PlosMedicine. (DOCX)
