# Supplementary material for: Clinical and neonatal outcomes of patients of different ages following transfer of thawed cleavage embryos and blastocysts cultured from thawed cleavage-stage embryos
Source: PLoS One. 2018 Nov 26;13(11):e0207340. doi: 10.1371/journal.pone.0207340 (PMC6261106; doi:10.1371/journal.pone.0207340)
Supplement: S2 File — (DOCX) [file pone.0207340.s002.docx]

STROBE Statement—checklist of items that should be included in reports of observational studies

|  | Item No. | Recommendation | Page  No. | Relevant text from manuscript |
| --- | --- | --- | --- | --- |
| **Title and abstract** | 1 | (a) Indicate the study’s design with a commonly used term in the title or the abstract | 2 | retrospective study |
|  |  | (b) Provide in the abstract an informative and balanced summary of what was done and what was found | 2 | blastocysts cultured from thawed cleavage-stage embryos are associated with fewer miscarriages and better perinatal outcomes than cleavage-stage FET；in this study we aimed to identify the optimal FET strategy in young and advanced age women who undergo FET |
| Introduction | | | |  |
| Background/rationale | 2 | Explain the scientific background and rationale for the investigation being reported | 4 | Since the first successful case of clinical pregnancy established using frozen embryos in the 1980s, frozen-thawed embryo transfer (FET) has become a routine procedure in [assisted](../../AppData/Roaming/æˆ‘çš) [reproductive](../../AppData/Roaming/æˆ‘çš) [technology](../../AppData/Roaming/æˆ‘çš) (ART) . Cryopreservation techniques and the developmental potential of frozen-thawed embryos have considerably improved since then, and the clinical pregnancy rates and some clinical outcomes associated with FET are even higher than those of fresh embryos . There were no safety issues in children born from ART using frozen cycles compared to those born from ART using fresh cycles |
| Objectives | 3 | State specific objectives, including any prespecified hypotheses | 5 | In this study, we approached the abovementioned problems by studying the clinical and perinatal outcomes of young patients (<35 years) and advanced age patients (≥35 years). Our study aims to identify a suitable FET strategy for infertile women who undergo frozen cleavage-stage embryo transfer associated with the best outcomes. |
| Methods | | | |  |
| Study design | 4 | Present key elements of study design early in the paper | 5 | retrospective cohort study |
| Setting | 5 | Describe the setting, locations, and relevant dates, including periods of recruitment, exposure, follow-up, and data collection | 5 | we traced infertile couples who had completed at least one FET cycle from January 2010 to December 2015. |
| Participants | 6 | (a) Cohort study—Give the eligibility criteria, and the sources and methods of selection of participants. Describe methods of follow-up  Case-control study—Give the eligibility criteria, and the sources and methods of case ascertainment and control selection. Give the rationale for the choice of cases and controls  Cross-sectional study—Give the eligibility criteria, and the sources and methods of selection of participants | 6 | The inclusion criteria were as follows: 1) maternal age, 20–42 years; 2) ≥3 frozen embryos; 3) frozen embryos are at the cleavage stage; 4) the first FET cycle after a previous fresh cycle. The patients were screened according to the following exclusion criteria: 1) oocyte donation cycles; 2) mixed cleavage-/blastocyst-stage embryo transfer cycles; 3) preimplantation genetic diagnostic/preimplantation genetic screening (PGD/PGS) cycles. |
|  |  | (b) Cohort study—For matched studies, give matching criteria and number of exposed and unexposed  Case-control study—For matched studies, give matching criteria and the number of controls per case | 6 | The clinical and neonatal outcomes of these two FET strategies were compared in young (<35 years) and advanced age (≥35 years) women. In our study, the thawed embryo transfer strategy to be used was decided depending on the patients’ decision. Before FET, we spoke with the patients who froze their embryos at the cleavage stage and informed them of the possible circumstances when transferring cleavage-stage embryos or blastocysts cultured from thawed cleavage-stage embryos. |
| Variables | 7 | Clearly define all outcomes, exposures, predictors, potential confounders, and effect modifiers. Give diagnostic criteria, if applicable | 10 | The implantation rate was calculated as the ratio of the number of observed embryo heartbeats to the number of transferred embryos. Clinical pregnancy was confirmed by an ultrasound conducted 4 weeks after the ET. The clinical pregnancy rate per transfer cycle was calculated as the number of clinical pregnancy cycles to the number of ET cycles. The pregnancy rate per thaw cycle was the number of clinical pregnancy cycles divided by the number of thaw cycles. The live birth rate per ET cycle was defined as the number of deliveries resulting in live births divided by the number of ET cycles. The live birth rate per thaw cycle was the number of deliveries resulting in live births cycles divided by the number of thaw cycles, delivery of a multiple pregnancy was considered a single live birth. The early/late miscarriage rate was calculated as the number of early/late miscarriage cycles to the number of clinical pregnancy cycles. Gestational age was defined as the number of completed weeks of gestation and calculated using the formula “(pregnancy end date – embryo transfer date) + 16 days” for cleavage embryo transfer and “(pregnancy end date – embryo transfer date) + 19 days” for blastocyst transfer. |
| Data sources/ measurement | 8* | For each variable of interest, give sources of data and details of methods of assessment (measurement). Describe comparability of assessment methods if there is more than one group | 7 | In strategy A, two cleavage-stage embryos with the highest grades were thawed and cultured in G2.5 media (Vitrolife) for 1–2 h. Embryos were considered to have survived and suitable for transfer if at least half of their blastomeres were intact after culture. In strategy B, all cleavage-stage embryos were thawed and the surviving embryos were cultured in G2.5 media (Vitrolife) up to day 5 in the presence of 6% CO2, 5% O2, and 89% N2 in a mini-incubator (Cook). Blastocyst morphology was evaluated on day 5 using Gardner’s grading system . Blastocysts that reached ≥3 BC were considered suitable for transfer, and those that reached ≥4 BB were considered to be of high quality. Only one high-quality blastocyst or at most two suitable blastocysts were transferred per FET cycle. The remaining suitable blastocysts were frozen again. |
| Bias | 9 | Describe any efforts to address potential sources of bias | 11 | Logistic regression analysis was used to control for potential confounders, including age, infertility types, endometrial preparation, and endometrial thickness, and to evaluate any potential effects (strategy A vs. strategy B in young women and advanced age women). |
| Study size | 10 | Explain how the study size was arrived at | 5 | A total of 16,387 frozen-thawed embryo cycles were incorporated in the study, including 15,408 embryo transfer cycles and 979 cancelled cycles in these transfer cycles. A total of 30,002 embryos were transferred. Patients who perform cryopreservation embryo transfer were divided into thawed cleavage-stage embryo transfer (Strategy A, n = 13,041), and transfer of blastocysts cultured from thawed cleavage-stage embryos (Strategy B, n = 3,346). |

Continued on next page

| Quantitative variables | 11 | Explain how quantitative variables were handled in the analyses. If applicable, describe which groupings were chosen and why | 5 | In this study, we included papers involving two FET strategies: thawed cleavage-stage ET (strategy A) or transfer of blastocysts cultured from thawed cleavage-stage embryos (strategy B). The clinical and neonatal outcomes of these two FET strategies were compared in young (<35 years) and advanced age (≥35 years) women. |
| --- | --- | --- | --- | --- |
| Statistical methods | 12 | (a) Describe all statistical methods, including those used to control for confounding | 8 | Statistical analysis was performed using Statistical Package for Social Sciences software version 19.0 (SPSS). Before analyses, a normality test was conducted for continuous variables. Normally distributed data were expressed as means ± SD, and data with non-normal distribution were expressed as median (range). Differences between the strategies were compared using the independent Student’s t test in normally distributed data, and Mann-Whitney test was applied to compare differences in non-normally distributed data. Categorical variables were presented as percentages. The χ2 test was used for categorical variables, and Fisher’s exact test was used if necessary. A P value of <0.05 was considered statistically significant. |
|  |  | (b) Describe any methods used to examine subgroups and interactions | N/A | No subgroups and interactions |
|  |  | (c) Explain how missing data were addressed | N/A | No missing data |
|  |  | (d) Cohort study—If applicable, explain how loss to follow-up was addressed  Case-control study—If applicable, explain how matching of cases and controls was addressed  Cross-sectional study—If applicable, describe analytical methods taking account of sampling strategy | N/A | No loss to follow-up |
|  |  | (e) Describe any sensitivity analyses | N/A | No sensitivity analyses |
| Results | | | | |
| Participants | 13* | (a) Report numbers of individuals at each stage of study—eg numbers potentially eligible, examined for eligibility, confirmed eligible, included in the study, completing follow-up, and analysed | 5 | A total of 16,387 frozen-thawed embryo cycles were incorporated in the study, including 15,408 embryo transfer cycles and 979 cancelled cycles in these transfer cycles. |
|  |  | (b) Give reasons for non-participation at each stage | N/A | No non-participation |
|  |  | (c) Consider use of a flow diagram | 5 | Figure 1 presents a flowchart illustrating the study design. |
| Descriptive data | 14* | (a) Give characteristics of study participants (eg demographic, clinical, social) and information on exposures and potential confounders | 9 | Patient characteristics |
|  |  | (b) Indicate number of participants with missing data for each variable of interest | N/A | No missing data for each variable of interest |
|  |  | (c) Cohort study—Summarise follow-up time (eg, average and total amount) | 8 | As part of the routine follow-up, couples were contacted by phone to obtain neonatal information, including their dates of birth, birth weight, gender, birth defects, and neonatal diseases. |
| Outcome data | 15* | Cohort study—Report numbers of outcome events or summary measures over time | 9 | A total of 16,387 frozen-thawed embryo cycles were incorporated in the study, including 15,408 embryo transfer cycles and 979 cancelled cycles in these transfer cycles. |
|  |  | Case-control study—Report numbers in each exposure category, or summary measures of exposure | N/A | Not Case-control study |
|  |  | Cross-sectional study—Report numbers of outcome events or summary measures | N/A | Not Cross-sectional study |
| Main results | 16 | (a) Give unadjusted estimates and, if applicable, confounder-adjusted estimates and their precision (eg, 95% confidence interval). Make clear which confounders were adjusted for and why they were included | 15 | Table3 |
|  |  | (b) Report category boundaries when continuous variables were categorized | 8 | Preterm and post-term births were deﬁned as deliveries before 37 or after 42 completed weeks of gestation. Neonates were categorized by birth weight: normal (2,500–4,000 g), very low birth weight (<2,500 g), and macrosomia (>4,000 g). |
|  |  | (c) If relevant, consider translating estimates of relative risk into absolute risk for a meaningful time period | N/A | No relative risk |

Continued on next page

| Other analyses | 17 | Report other analyses done—eg analyses of subgroups and interactions, and sensitivity analyses | N/A | No analyses of subgroups and interactions, and sensitivity analyses |
| --- | --- | --- | --- | --- |
| Discussion | | | | |
| Key results | 18 | Summarise key results with reference to study objectives | 13 | The results of our study revealed that thawed cleavage-stage embryo transfer was more suitable for women <35 years of age, while transfer of blastocysts cultured from thawed cleavage-stage embryos was found to be more suitable for women ≥35 years of age. |
| Limitations | 19 | Discuss limitations of the study, taking into account sources of potential bias or imprecision. Discuss both direction and magnitude of any potential bias | 17 | the results of the present study should be regarded with caution due to its limitations, primarily the retrospective design and confounding factors. |
| Interpretation | 20 | Give a cautious overall interpretation of results considering objectives, limitations, multiplicity of analyses, results from similar studies, and other relevant evidence | 17 | In future, randomized controlled trials with a large sample size should be carried out to verify the findings of this study. |
| Generalisability | 21 | Discuss the generalisability (external validity) of the study results | 17 | This study validates existing data and provides useful information for counseling patients undergoing FET to select a suitable strategy to improve their chances of a healthy and safe pregnancy and birth of a healthy child. |
| Other information | |  | | |
| Funding | 22 | Give the source of funding and the role of the funders for the present study and, if applicable, for the original study on which the present article is based | 1 | This work was supported by the National Natural Science Foundation of China [grant number 31401069] and the Natural Science Foundation of Hunan Province, China [grant number 2017JJ2327], and the National Key Research and Development Program of China [grant number 2016YFC1000200] |

*Give information separately for cases and controls in case-control studies and, if applicable, for exposed and unexposed groups in cohort and cross-sectional studies.

**Note:** An Explanation and Elaboration article discusses each checklist item and gives methodological background and published examples of transparent reporting. The STROBE checklist is best used in conjunction with this article (freely available on the Web sites of PLoS Medicine at http://www.plosmedicine.org/, Annals of Internal Medicine at http://www.annals.org/, and Epidemiology at http://www.epidem.com/). Information on the STROBE Initiative is available at www.strobe-statement.org.
